# The changing face of clinical trials in the personalized medicine and immuno-oncology era: report from the international congress on clinical trials in Oncology & Hemato-Oncology (ICTO 2017)

**DOI:** 10.1186/s13046-017-0668-0

**Published:** 2017-12-28

**Authors:** Talia Golan, Michele Milella, Aliza Ackerstein, Ranaan Berger

**Affiliations:** 1 Oncology Institute, Sheba Medical Center, Emek HaEla St 1, Tel Hashomer, Ramat Gan, Israel; 2 Sackler Faculty of Medicine, Tel Aviv University, Tel Aviv, Israel; 3 Division of Medical Oncology 1, Regina Elena National Cancer Institute, via Elio Chianesi 53, 00144 Rome, Italy

**Keywords:** Oncology, Hematology, Clinical trials, Immuno-oncology, Targeted therapy, Personalized medicine, Biostatistics, Endpoints, Methodology

## Abstract

In the past decade, the oncology community has witnessed major advances in the understanding of cancer biology and major breakthroughs in several different therapeutic areas, from solid tumors to hematological malignancies; moreover, the advent of effective immunotherapy approaches, such as immune-checkpoint blockade, is revolutionizing treatment algorithms in almost all oncology disease areas. As knowledge evolves and new weapons emerge in the “war against cancer”, clinical and translational research need to adapt to a rapidly changing environment to effectively translate novel concepts into sustainable and accessible therapeutic options for cancer patients.

With this in mind, translational cancer researchers, oncology professionals, treatment experts, CRO and industry leaders, as well as patient representatives gathered in London, 16-17 March 2017, for *The International Congress on Clinical Trials in Oncology and Hemato-Oncology (ICTO2017)*, to discuss the changing face of oncology clinical trials in the new era of personalized medicine and immuno-oncology. A wide range of topics, including clinical trial design in immuno-oncology, biomarker-oriented drug development paths, statistical design and endpoint selection, challenges in the design and conduct of personalized medicine clinical trials, risk-based monitoring, financing and reimbursement, as well as best operational practices, were discussed in an open, highly interactive format, favoring networking among all relevant stakeholders. The most relevant data, approaches and issues emerged and discussed during the conference are summarized in this report.

## Introduction

Developing novel, safer treatments that may be curative for many individuals living with cancer depends on the clinical and regulatory success of the newest treatments - including promising developments focused on targeted therapy/immunotherapy combinations and immune checkpoint blockade therapies, which are demonstrating that immunity is key to long-term responses in many types of cancer [1, 2]. Personalized and precision medicine approaches, on the other hand, are also fostering a major change in the way we currently practice oncology and hemato-oncology, leading to a rapid change of treatment paradigms and algorithms, resulting in a wealth of new treatment options and accelerated discovery pathways. Adapting to such a novel and rapidly evolving scenario is challenging and requires all stakeholders (governments, research industry, biomedical community, pharmaceutical industry, patient groups, and regulatory bodies) to make a common and concerted effort to make as many new and effective treatments as possible available to cancer patients who need them, in the safest, fastest, and most effective way [3, 4]. In that respect, the success rate in developing new oncology drugs has historically been surprisingly low as compared to other areas of medicine, particularly in late phases of development, highlighting complexities and challenges in the new era of personalized/precision medicine and immune-oncology and suggesting the need to identify new models and paths to a fast, successful, and cost-effective drug development [5, 6]. Rigorous design and evaluation of clinical trials to assess efficacy and safety in patients and to prioritize development paths are critical to success; new molecular entity selection, protocol development, companion diagnostics, patient population, principal investigator/site selection and management, and monitoring strategies are key areas on which to focus to establish a foundation for the successful execution of both early and late phase oncology and hemato-oncology trials. With this in mind, translational cancer researchers, oncology professionals, treatment experts, contract research organizations (CRO) and industry leaders, as well as patient representatives, gathered in London, 16-17 March 2017, for *The International Congress on Clinical Trials in Oncology and Hemato-Oncology (ICTO2017)*, to discuss the changing face of oncology clinical trials in the new era of personalized medicine and immuno-oncology.

## The immunotherapy revolution

The landscape of early phase oncology trials is evolving to adapt to novel immune-therapies [1, 2] and to improve the efficiency of drug development. The session opened with a keynote presentation on the immune system and its interaction with cancer cells by Dr. Gal Markel, Ella Lemelbaum Institute of Melanoma, Sheba Medical Center. Dr. Shilpa Gupta, University of Minnesota Minneapolis, USA, discussed novel immunotherapeutic combination in clinical trials. Dr. Talia Golan, Sheba Medical Center, discussed new approaches in immunotherapy early phase drug development.

The introduction of a new class of drugs and changing clinical trial landscape has proposed challenging aspects to the treating physician. Traditional definitions, including dose-limiting toxicity (DLT: toxicity that is considered severe enough to limit further dose escalation) and maximum tolerated dose (MTD: this is the dose where no more than 30% of treated patients will experience DLT), have not changed. However, these definitions were based on the empirical linear relationship between dose, efficacy and toxicity seen in chemotherapies and some molecular targeted agents (MTA). This is one of the major changes in early drug development in immuno-oncology (I-O). Since the majority of the phase I trials to date in immune-stimulatory monoclonal antibodies have not reached DLT dose, recommended phase II dose (RP2D) is usually based on maximum administered dose or PK data. Enrollment of patients at the end of a Phase I trial of a particular tumor type and/or genetic subtype entitled expansion cohorts. Due to significant early efficacy signals in I-O large expanded cohorts are being utilized in Phase I trial designs. Expansion cohorts traditionally included 10-15 patients are expanding significantly to include 100-1000 patients [7, 8]. Early phase I programs need to be well integrated within subspecialty clinics to facilitate dose expansions. Furthermore, later phase clinical trial units participating in the expansion cohorts need to have the infrastructure to access and capture early and late I-O related toxicities, since these drugs are still at early initial safety assessments. Dramatic responses seen in early phase clinical trials have changed the traditional phase I/II/III approach. Furthermore, complexity has increased substantially based on different models of immune response.

## Clinical trials and companion diagnostics in hematology and solid tumor oncology

For many years hematology has been leading the way towards precise molecular characterization, patient selection, and therapy monitoring in clinical trials and routine clinical practice alike. According to Prof. Robin Foà, Head of Hematology at the “Sapienza” University of Rome, this has profoundly impacted on the management of both malignant and benign hematologic disorders over the past 20 years. A broad and integrated characterization at diagnosis in onco-hematology, achieved through central processing, strict laboratory validation, and standardized methodologies incorporated in clinical trials, has led to more accurate diagnostic work-up, precise definition of disease sub-entities, biologically-based prognostic stratification and identification of molecular markers for minimal residual disease (MRD) monitoring, implementation of effective targeted therapies, and identification of novel potential therapeutic targets in all age groups, including the elderly. This has resulted in an impressive improvement in overall survival (OS), now exceeding 90% at 5 years, among children with acute lymphoblastic leukemia (ALL) who were enrolled in Children’s Cancer Group and Children’s Oncology Group Clinical Trials between 1968 and 2009 [9]. In acute promyelocytic leukemia, the identification of specific genetic lesion(s), which imply exquisite sensitivity to targeted differentiating therapy, have led to the development of chemotherapy-free treatment approaches, such as the combination of all-*trans* retinoic acid (ATRA) and arsenic trioxide (ATO) [10, 11], endowed with 100% complete response (CR) rate and no toxic deaths or induction of resistance, particularly when MRD is monitored by sensitive techniques, such as RT-PCR, as in the Italian AIDA trial [12]. Other examples include chronic myeloid leukemia (CML), in which life-expectancy of affected patients now approaches that of the general population after the introduction of different generations of tyrosine kinase inhibitors (TKI) [13] and the development of molecularly-based MRD monitoring strategies [14, 15]; similarly, molecularly-based prognostic stratification and combined TKI-based treatment strategies have led to the implementation of chemotherapy-free induction regimens endowed with 97-100% CR rates and no induction deaths in Philadelhpia + ALL [16], suitable for the safe and effective treatment even of patients aged >90 years. In chronic lymphocytic leukemia (CLL), molecular characterization by high-throughput technologies are redefining risk groups and paving the way for truly individualized treatment strategies, based on the availability of a wealth of drugs targeted to specific molecular aberrations [17]; similarly, the discovery of pathogenic BRAF mutations in hairy cell leukemia (HCL) has had diagnostic and therapeutic implications [18]. The above advances that have translated in developments from bench to the bedside are only possible through: 1) adequate and accessible laboratories; 2) access to drugs; 3) close interaction between the clinic and the laboratories (including training and availability of physician-scientists); 4) multicenter networks/collaborative groups; 5) central handling and banking of biological material; 6) national and international collaborations; 7) dedicated and motivated individuals/teams; 8) close collaboration between academia and the pharmaceutical industry; 9) adequate funding. However, as Prof. Foà noted at the end of his talk, the world is vast and local realities are very different in terms of patient populations (life expectancy, age distribution), disease epidemiology, diagnostic, prognostic and therapeutic possibilities, drug availability and regulations, key issues of accessibility (to technologies and drugs) and sustainability for patients and countries; right now, cooperative study groups (and thus efficient and effective clinical trials) are, unfortunately, for the lucky few.

In general, drug development in oncology has not been efficient in coping with increasing complexity, as noted by Dr. Michele Milella, from the Regina Elena National Cancer Institute in Rome. Likelihood of approval (i.e. the probability of reaching regulatory approval from the current phase, expressed as a percentage - LOA) and phase success rates (i.e. the number of drugs that moved from one phase to the next phase divided by the sum of the number of drugs that progressed to the next phase and the number of drugs that were suspended) are lower in oncology than in other therapeutic areas (LOA from phase *I* < 6%) and in solid tumors as opposed to hemato-oncology (approximately 50% in each phase of development) [5]. One way to overcome such inefficiency in current drug development in solid tumors would be to implement the use of biomarker-driven strategies and paying close attention to the results of phase I/II proof-of-concept (POC) studies; indeed, in a recent analysis of 80 drug programs, approximately 75% of the drugs developed with biomarker-driven studies and/or with a positive POC achieved regulatory approval, as opposed to 15-30% of those that had no putative biomarker or for which POC studies were not performed or had negative results [6]. For this very reason, regulatory agencies, such as FDA, are putting the accent on the importance of co-developing novel drugs or therapeutic products together with “companion diagnostics” (CDx); according to the FDA definition, a CDx test is “a medical device that provides information that is essential for the safe and effective use of a corresponding therapeutic product” and play an important role “in determining that the safest and most effective treatments are promptly delivered to patients living with serious and life-threatening diseases” (Alberto Gutierrez, Office of In Vitro Diagnostic Device Evaluation and Safety of the FDA’s Center for Devices and Radiological Health, on Vysis ALK Break Apart FISH Probe Kit by Abbott for selecting patients candidate to Xalkori®) [19]. Although the pharmaceutical industry is also starting to recognize the importance of biomarker identification and CDx co-development, such drug development paradigm is still grossly underutilized: indeed, from 1998 to 2016 only 16/167 drugs approvals (9.6%) were based/required a specific CDx; over the past 18 years the cost-to benefit ratio for the great majority of the approved drug (90.4%) was felt not to be in favor of developing a CDx test and the successful strategy was to gain approval for an unsegmented indication, with lower response rate (RR) and lower average progression-free survival (PFS) and OS benefits [20, 21]. In the current scenario, identifying and validating relevant prognostic/predicting biomarkers and developing cost-effective CDx is a complex task, raising a number of important issues, from tissue procurement, to assay validation, and regulatory approval [22], that were approached in more detail in a subsequent session focusing on precision medicine (see below). As biological complexity increases, due to the enormous amount of information generated by extensive, *omics-based* genetic and epigenetic profiling of individual tumors, new challenges arise in the way we design and conduct modern clinical trials, especially since in most instances we do not deal anymore with the equation one mutation, one aberrant gene product, one target. Adaptive designs and “umbrella” trials are increasingly being used to cope with increased biological complexity and the need to rapidly test multiple hypotheses, drugs, biomarkers at once, as exemplified by the BATTLE program in lung cancer or by the I-SPY program in breast cancer, to name a few [23, 24]. As discussed in the previous section, the advent of immune-oncology adds a further level of complexity, particularly in light of its peculiar mechanism of action, requiring innovative trial designs and endpoints, and of the current lack of solid, validated biomarkers/CDx [25, 26].

Finally, Prof. Aldo Scarpa from the University of Verona and the ARC-NET Research Center elaborated on what modern molecular pathology can do to provide oncologists with what he defined as an “actionable diagnosis”. Recognition of the profound molecular heterogeneity of cancers [27], on one hand, and the possibility to group different cancers into discrete “cancer biotypes”, on the other, has prompted a major paradigm shift, from highly selective molecular testing (as it currently happens for EGFR and ALK testing in non-small cell lung cancer - NSCLC) towards unbiased whole-genome/whole-transcriptome testing [28]. The latter requires totally new approaches to the prioritization and handling of diagnostic material, particularly for advanced patients who do not usually undergo surgery and for whom only limited biotic material is available, in that it requires frozen samples and it is profoundly influenced by the cellular composition/quality of the sample [29]; moreover, such approach also requires a heavy bioinformatics workload and long turnaround times, that are not always compatible with, for example, its use for clinical trial selection. As technology and bioinformatics evolve, an alternative possibility, proposed by several collaborative groups, is multiplex testing: this allows for simultaneous multigene and multiplexed mutation and RNA profiling/fusion transcript detection, as well as genomic copy number profiling, on a focused panel of genes/transcripts [28]; in the current scenario, this has several advantages in that it can use formalin-fixed paraffin-embedded (FFPE) tissue, thus minimally influencing routine tissue handling, significantly cutting costs, and simultaneously allowing for standard histopathological examination. Targeted sequencing approaches have, for example, recently shown potential for better selection of patients for EGFR-TKI treatment, looking at a relatively limited panel of genes that can be simultaneously mutated in addition to EGFR in a proportion of EGFR-mutant NSCLC, resulting in different probability of response [30]. Targeted panels currently in development, such as the Oncomine assay and disease-specific panels being developed within the International Cancer Genome Consortium (ICGC), are able to detect single nucleotide variations, amplifications (including focal amplifications), translocations, deletions and copy number variations, paving the way for what Prof. Scarpa defined “next generation histopathology”, where molecular alterations are mapped to specific histological patterns. As a practical example, he showed the development of the HR-1 targeted next generation sequencing (NGS) kit for the detection of BRCA somatic and germline mutations in a series of 47 high-grade serous ovarian cancers [31]. Other examples included molecular subtyping of pancreatic adenocarcinoma samples [32, 33], using five different custom targeted NGS panels, encompassing, among others, stromal pathways, such as the transforming growth factor (TGF)-β pathway, and integrating the characterization of the tumor stroma, using newer histopathological techniques, such as DEP-array single cell sorting and low pass whole genome sequencing. Not only will these techniques becoming part of the routine diagnostic approach to selected cancers, but they will also greatly contribute to prognostic/predictive assessment and possibly to non-invasive disease monitoring (for example using liquid biopsy, see below), both within and outside clinical trials.

## Methodology and patients’ perspectives in setting the right endpoints

The sesssion began with Matt Ellefson presenting a patient’s perspective on clinical trials in cancer. Next, Dr. Diana Giannarelli discussed the complexity of traditional survival end points and suggested alternative end points, as a tool to further promote the development of new therapeutics in oncology. Finally, the session was ended with an educational discussion of new approaches in designing a pivotal trial in oncology by Andrew Stone, from Stone Biostatistics.

From the perspective of Matt Ellefson, founder of SURVIVEiT®, a global community of survivors, doctors, experts, and allies who provide hope, knowledge, and resources crucial to surviving cancer, clinical trials serve a vital role in the development and advancement of life-saving medicines for the treatment of cancer. Without clinical trials, there is no progress. Matt Ellefson, a seven-year survivor of advanced lung cancer, discussed his cancer care journey, which includes two clinical trials. Matt discussed why he feels clinical trials are underutilized today and why the makers of life-saving medicines have difficulty meeting clinical trial participation goals. From his perspective, there is still a stigma that clinical trials are only an experimental option for patients that are nearly dead. Matt claims that this couldn’t be further from the truth and that today’s clinical trials are not only safe, but offer patients with access to tomorrow’s medicines [34, 35].

Diana Giannarelli from the Biostatistical Unit at the Regina Elena National Cancer Institute in Rome, discussed endpoints in clinical trials in oncology. When designing a clinical trial the choice of endpoint is crucial. OS is often considered as the gold standard because of it certainty, because it is reliable and in general beneficial for patients. Dr. Giannarelli discussed different statistical instruments that are being used to overcome problems that occur during follow up measurements. One example is when time to event of a particular patient is being ‘censored’ and the available information consists only of a lower bound for its actual event time. Another problem is that of competing events to the actual endpoint (death from a car accident for example). Conventionally, survival analysis is performed by assuming the survival distribution as exponential and the hazards as proportional over time. With these assumptions, the summarized median survival may be reliably and consistently estimated before all survival events are actually observed. However, the different mechanism(s) of action of new drugs (particularly immune-oncology drugs), a landscape of many therapeutic alternatives and different clinical scenarios may undermine such an approach [36]. She suggested that the possibility of crossover, mandatory for ethical aspects, could confound the ‘real’ effect of the experimental therapy and the adjustment for this opportunity needs to be implemented in the statistical analysis of results. Moreover, different metrics can be used, such as restricted mean survival time, cure fraction and survival rates at pre-specified timepoints and should be considered as alternative endpoints, especially in immunotherapeutic studies in which a delayed effect is reasonably expected. Very recently the use of these metrics is more than just suggested and an increasing number of paper are proposing its use in designing clinical trials [37]. Finally, Dr. Giannarelli addressed the study conducted by Hotta and others on 34 randomized clinical trials (RCT) for which they examine the relationship between hazard ratios (HR) for PFS and for OS. The HR for PFS and OS are not correlated when considering all the studies, but when separating for whether crossover was allowed or not, the linear relationship comes out. In this study, it was pointed out that this relationship depends on post-progression survival that is the time between progression and death. If the distribution of survival after progression is the same in both treatment arms then the benefit in survival is the same as the benefit in delaying progression [38, 39]. Additionally, survival times can be split in many subsequent times to event(s), corresponding to different lines of therapy; this might be useful to refine phase III clinical trial design: one example is to define PFS2 as the time to progression to the subsequent therapy or death, that is appearing in many trials design. Dr. Giannarelli concluded that with so many effective new drugs, an alternative way of overcoming crossover problems could be to design strategy trial using ‘comprehensive’ time to events trying to lessen post-progression survival out of the trial [40, 41].

Dr. Andrew Stone (Stone Biostatistics) presented his professional vision in a presentation entitled “Keys to successful pivotal trial design in oncology”. There are a multitude of questions to consider when designing a pivotal trial in oncology in order for a product to succeed. Dr. Stone’s presentation gave an overview discussion of the essential questions we need to answer to, focusing on: 1) cohorts size: what statistical power do we need and how does it influence cohort size? How large should the trial be based on clinical precedents, data on your own compound, including its uncertainty, and whether the effect is immediate? How to design a trial when the effect of a therapy is delayed and how to adapt calculations [42]; 2) what should be the primary endpoint(s)? PFS versus OS: Dr. Stone pointed to the fact that understanding the relationship for PFS and OS might be helpful; for example, that relationship is absent in IO trials and one should take it into account and not have PFS as the primary endpoint [43]; 3) options to hedge your bets when there is >1 endpoint, experimental arm or population, or if you’re not sure whether your drug will only work in a biomarker defined subgroup or in all patients [44, 45]. Dr. Stone pointed out that a good strategy to split your alpha: you can ‘spend’/split your alpha whatever way you want, as long as it is pre-specified. This is a more reasonable solution than having to repeat the trial in the subgroup itself. When there are 2 experimental arms (mono and combo) and 2 key endpoints, Dr. Stone suggested to test which endpoints, in which order, and If win on one then can recycle to others. Perhaps the most important point is that we should not “mess a great design up with poor conduct of the trial”. We should be strict with completeness of data, trying to: 1) obtain PFS and OS events, regardless of whether the patient withdrew from therapy as long as patients have not withdrawn consent; 2) monitoring the trial as it proceeds, avoiding to just clean what finds its way on to the database and actively querying sites if data are missing; 3) using survival sweep to confirm status in all patients thought to be still alive [46].

## Challenges to *personalized medicine*

In a dedicated session, 4 presentations covered different aspects of personalized medicine. Dr. Baker presented the program of translational and clinical program at the University of Michigan Comprehensive Cancer Center (UMCCC) focusing on the launched joint project between its developmental therapeutics program and its translational and clinical research programs. The aim there is to advance novel UMCCC clinical candidates into early phase I, IB and II trials. Features of this program include genomic interrogation using the Michigan Oncology Sequencing Program MIOncoSeq, a 1700 gene deep sequencing program developed in-house; He presented some data from the first 500 metastatic patients enrolled in the program where 11% had a newly identified pathogenic germline alteration identified as a result of sequencing [47, 48]. Dr. Baker indicated that they are also using monitoring of cell-free tumor DNA in plasma, as a liquid biopsy approach to monitor response to treatment over time. The developmental therapeutics program includes several successful laboratory discoveries, which already succeeded in developing 8 compounds which are undergoing lead optimization. Dr. Baker presented six drugs from these discovery programs that are currently in the clinic (ATR101-ACAT; SM406-cIAP; Mi773-MDM2; SM1387-cIAP/XIAP; AA115-MDM2 and BM1252-BCL2/XL). Until now the model used was to seek a partnering relationship with preclinical data; the alternative new model seeks to partner with drug companies after the successful completion of phase 1 trials: Dr. Baker presented the dedicated phase 1 research unit with capabilities including: timed blood, urine, PK/PD sampling; investigational drug accountability/compliance; and clinical trials bank processing, storage and shipping. He concluded that the UMCCC team-based strategy is to address the care of the patients and the science of the drugs.

Dr. Raanan Berger from the Sheba Medical Center in Israel discussed the issue of how realistic is tissue biopsy in clinical trials. He first defined the difference between clinical and research biopsies: clinical biopsies are obtained for diagnosis and delineating markers of response to targeted therapy; research biopsies are being done as part of clinical trial protocol with the intent to answer a specific, scientific question (or several questions) through the use of correlative assays. Regarding safety of research biopsy, the data is limited, and the reporting of adverse events associated with the collection of research tissue is not standardized. However, most patients who undergo mandatory research biopsies tolerate the procedure well [49, 50], with anxiety reported in 30% of trial participants. Prospective clinical trial designs require the sampling of fresh tumor biopsy tissue for exploring resistance mechanisms: in his presentation, Dr. Berger discussed molecular ‘two-stage’ exploratory design for patients that have defined actionable mutations that match the investigational drug [51]. He then presented a study published by Dr. Ratain’s group, analyzing the impact of post-treatment biopsies in phase I clinical trials. The estimation is that at present post-treatment biopsies in studies are almost mandatory. Just 5 of the 72 studies that was found in the literature produced a statistically significant biomarker result that was cited in subsequent publications. Just 2 of these were cited by research groups other than those performing the original study. Dr. Berger cited Dr. Ratain saying that “The bottom line is that we need to do fewer studies with post-treatment biopsies, and when we do decide to do them, we need to do a much better job” [52]. Finally, Dr. Berger discussed Phase 0 clinical trials, which is a first in human trial with limited number of patients aiming to generate data to enhance efficiency and to increase chance of success of subsequent development of the agent [53]. He concluded his talk with ethical issues need to be considered when designing a trial that include research biopsy as part of it [54].

Dr. Giulia Siravegna, from the Candiolo Cancer Institute, demonstrated how to overcome the difficulties in accessing tissue samples from resistant colorectal cancer by using liquid biopsies for our understanding of the heterogeneous molecular bases of secondary resistance to EGFR blockade [55]. The aim of her study was to demonstrate the way liquid biopsies could be exploited to genotype colorectal cancers using circulating tumor DNA (ctDNA), to compare molecular profiles obtained in tissue and ctDNA, identify molecular mechanisms of resistance to EGFR blockade in CRC patients and to monitor clonal evolution during therapy. She addressed the point that tissue-based tumor profiles are subjected to sampling bias, provide only a snapshot of the tumor heterogeneity and cannot be obtained repeatedly. Dr. Siravegna presented her data on 100 CRC patients with 200 samples analyzed (plasma samples and matched tissue DNA) showing 97% plasma-tissue concordance [56]. She presented data showing that ctDNA was used to genotype colorectal tumors and track clonal evolution during treatment with anti-EGFR antibodies, identifying alterations underlying primary and acquired resistance in KRAS, NRAS, MET, ERBB2, EGFR and MAP2K1 genes. Additionally, it was found that mutant KRAS clones that emerged during EGFR blockade declined upon withdrawal of anti-EGFR antibodies, indicating that clonal evolution continues beyond clinical progression [57, 58]. ctDNA profiles of individuals who benefit from multiple challenges with anti-EGFR antibodies exhibit pulsatile levels of mutant KRAS, providing a molecular explanation for the efficacy of rechallenge therapies based on EGFR blockade. Remarkably, ctDNA analyses were instrumental to show that responses to targeted therapies can be driven by distinct resistance mechanisms arising within separate tumor lesions in the same patient [59]. As more trials evaluating targeted therapy strategies designed to overcome specific acquired resistance mechanisms enter the clinic, genomic results from single-tumor biopsies should be interpreted with caution. By contrast, liquid biopsy approaches have the potential to detect the presence of simultaneous resistance mechanisms residing in separate metastases in a single patient and to monitor the effects of subsequent targeted therapies [60].

Dr. Raskin from the Sheba medical Center discussed his experience with thousands of patient encounters in clinical trials in order to demonstrate the practical use of image-based criteria in the context of randomized clinical trials. Medical oncology depends on an accurate assessment of tumor response to therapy. In the case of solid tumors, radiological imaging has assumed a dominant role in assessing response, because it can provide reliable and mostly objective measurements of tumor size. RECIST (Response Evaluation Criteria in Solid Tumours) is one of a family of systems with definitions and rules for measuring tumours and assigning them to discrete categories of response [61–64]. RECIST has emerged as the most common methodology for determining response to therapy in the field of clinical trials; its principles may be applied to the general practice of oncology, as well. The results of clinical trials are also of scientific merit, and, therefore, RECIST has become an accepted measure of efficacy both for regulatory approval and for advancing scientific knowledge regarding novel therapies. In this richly illustrated talk, Dr. Raskin examined RECIST and other similar image-based methodologies, with an emphasis on both objective and subjective criteria in assessing tumor response. Specific problem areas, such as non-measurable disease and “pseudoprogression” were addressed.

## Best practices in clinical trials

This session focused on the several different aspects of running clinical trials. Lucy Gilbert, McGill University, Canada discussed in depth the challenges in development and approval for diagnostic test in cancer [19].

Marta Arias-Salgado, Regional Head of Global Clinical Trials Operations from Merck, Sharp and Dohme (MSD) elaborated on the aspects of monitoring clinical trials in the era of immunotherapy. The complexity of clinical trials in oncology is well known; furthermore, the era of immunotherapy has significantly added further complexity to trials. Including additional protocol complexity: various companion tests, multiple vendors, numerous manuals and new data requirements. Different interpretation of traditional measures: RECIST vs RECIST 1.1, immune-related RECIST Criteria (irRC), and different flare patterns [60–64]. External environment complexity: number and magnitude of programs with continuous data generation impacting ongoing studies and programs [65–67].

Andréa Saud Martinez, Pharm.D., M.Sc., General Manager Atlantis Clinical Latin America gave a fascinating talk about conducting cancer clinical trials in Latin America with an emphasis on Brazil. The numerous opportunities included a large and rapidly rising population of largely trial naïve people. There are approximately 600 million people living in Latin America, which has a wide variety of races and ethnicities. Thus, providing a diverse patient population. In the whole region only two languages (Spanish and Portuguese) are used reducing the need to translate and produce materials in multiple languages. A growing list of sites practicing in accordance to ICH GCP guidelines. The physicians in Latin America are well-trained and individual countries in Latin America have made significant investments in improving and expanding healthcare for their citizens. Improved regulatory standards instituted in an effort to shorten clinical trial approval timeframes, thus providing an emerging and attractive market for pharmaceuticals. Challenges include: regulatory uncertainties with timelines frequently not met; ongoing logistical issues; cultural differences; limited cancer registries databases and lastly negative perception about clinical research by several government authorities [68].

## Risk-based monitoring (RBM) and financial sustainability

Lihi Bodenuk, Country Study Manager & Country Study Specialist from Roche Pharmaceuticals (Israel) discussed risk-based monitoring (RBM) in general and specifically in oncology clinical trials. Monitoring is an FDA-mandated process whereby the integrity of the clinical trial process is validated. Validation is a huge amount of work, and historically, hugely personnel-intensive. Specifically, monitoring is estimated to be about 1/3 of any prospective clinical trial operating budget. For an industry that spends over $30 billion on clinical trials, this means over $10 billion is spent on monitoring each year. Starting in 2011, the FDA issued a RBM guidance on modifications to this process, in an effort to encourage faster and less costly practices [69]. Five years later, there is still confusion around the options available to a sponsor for making this process more efficient. Each option deals with either reducing the scope of verification or making it easier on both the site and the sponsor. While RBM has been a popular discussion topic among the clinical research community for quite some time, sponsors and researchers have been slow to implement adaptive strategies. RBM moves away from the traditional approach of frequent on-site visits and 100% source data verification (SDV), toward a combination of activities, including centralized data collection and monitoring. The goal of traditional monitoring is to ensure patient safety and quality data. A properly designed RBM strategy can support and even enhance this practice while more efficiently using resources. There is growing concern from clinical study sites regarding the impact RBM processes will have on their workload, budgets and monitoring support. Given there is no one-size-fits-all approach to follow, study sites have expressed several areas of concern as to what RBM entails. While there are certainly broad implications for study sites when it comes to RBM, much of what they do will ultimately remain the same. More companies are now tailoring their monitoring plans to be proportionate to the identified risks of the trial. This presentation opened a discussion regarding the way responsibilities have shifted from the sponsor to the site, increasing workload and budget concerns, especially in oncology clinical trials. Sites must be prepared accordingly in order to meet these requirements [70].

Henrik Torp Nielsen, Head of Development Finance Oncology Region Europe, Novartis, and Ivana Matic, Site Ready & Support Lead – Global Collaboration, MSD, discussed financial aspects of an oncology clinical trial. This presentation described step by step how the sponsor builds the budget. The first step is to gather information from the sponsor protocol, IB manuals (lab, pharmacy, imaging coordinator), central vendor questionnaires, etc. The sponsor identifies study costs based on labor costs and researches rates for procedures. External budgets are negotiated with industry sponsors based on the fair market value. The external budget must cover all the costs included in the internal budget. Formats for sponsor budgets vary, as each sponsor requires their own format. There is a different budgeting format per region and per country according to fair market value. The budget is open for negotiation based on justification. The panel discussion reflected the varied interests of all relevant parties and raised important points as to the financial aspects of clinical trials. Currently, in budget determinations, sponsors allocate a budget per procedure without taking into account the work of medical staff and administration. Additionally, there is the additional cost to RBM sites which is not compensated, including the extra time of study coordinators, back office administration and data management. Furthermore, regardless of fair market value there are different budgets for the same procedures from the same sponsor.

Overall, the conference enabled key players in oncology clinical trials to engage in robust discussions and gave them a platform to raise specific concerns that they face in the financial operations of these clinical trials.

## Closing remarks

We are living an exciting time in cancer research, witnessing basic science discoveries and clinical/translational progress at an unprecedented pace, ultimately resulting in more and more effective therapeutic options and longer and healthier life expectancy for our patients. As we step into a new era of personalized/precision medicine, also involving novel approaches to harnessing the power of the immune system to control cancer growth from within the patient’s own body, we all face new challenges and need to adapt our practice in translational and clinical research to a rapidly changing, global scenario. Great successes achieved so far notwithstanding, there is plenty of room to learn from past errors, improve, and make the clinical research process faster and more cost-effective: indeed, we have to keep in mind that every failed trial is an unacceptable waste of time, resources, and, worst of all, patient lives. In the current scenario, we can’t afford not to do better (in economic terms as well) at bringing new and effective treatments to every patient’s bedside: science and clinical/statistical methodology need to develop common strategies and there is no way we can face the challenge of increasing biological complexity without evolving our way of thinking. The take home message that clearly emerged from the exciting 2 days we spent in London discussing the changing face of oncology clinical trials in the new era of personalized medicine and immuno-oncology is that we owe it to our patients (and to ourselves as clinical/translational researchers) to be more efficient at finding “the *right drug*, for the *right patient*, at the *right time*”. This can only be achieved with the coordinated contribution of all stakeholders: governments, research industry, biomedical community, pharmaceutical industry, patient groups, and regulatory bodies. With this in mind we are now looking forward to *The 2nd International Congress on Clinical Trials in Oncology and Hemato-Oncology (ICTO2018 -* http://www.icto2018.com), which will be held in Berlin, February 19-20, 2018. There, different oncology professionals, treatment experts, CRO, industry leaders and other experts in the cancer research field will convene again to discuss a range of topics: issues in the design and conduct of adaptive trials, operational hurdles, trials in hematology, agreements in oncology clinical trials, relevant preclinical models, case studies of precision medicine trials (such as an in-depth discussion on the design and conduct of the Precision Promise trial in pancreatic cancer), the role of collaborative research networks, ethics, patient advocacy and regulatory issues.

## Abbreviations

ALL: Acute lymphoblastic leukemia
ATO: Arsenic trioxide
ATRA: All-*trans* retinoic acid
CDx: Companion diagnostics
CLL: Chronic lymphocytic leukemia
CML: Chronic myeloid leukemia
CR: Complete response
CRO: Contract research organization
ctDNA: Circulating tumor DNA
DLT: Dose-limiting toxicity
FFPE: Formalin-fixed paraffin-embedded
HCL: Hairy cell leukemia
HR: Hazard ratios
ICGC: International Cancer Genome Consortium
I-O: Immuno-oncology
irRC: Immune-related RECIST Criteria
LOA: Likelihood of approval
MRD: Minimal residual disease
MTA: Molecular targeted agents
MTD: Maximum tolerated dose
NGS: Next generation sequencing
NSCLC: Non-small cell lung cancer
OS: Overall survival
PFS: Progression-free survival
POC: Proof-of-concept
RBM: Risk-based monitoring
RCT: Randomized clinical trials
RECIST: Response Evaluation Criteria in Solid Tumours
RP2D: Recommended phase II dose
RR: Response rate
SDV: Source data verification
TGF: Transforming growth factor
TKI: Tyrosine kinase inhibitors
UMCCC: University of Michigan Comprehensive Cancer Center
